# Case report: Preliminary response to tislelizumab plus S-1 in patients with metastatic gallbladder carcinoma: A report of five cases and a literature review

**DOI:** 10.3389/fimmu.2023.1144371

**Published:** 2023-03-20

**Authors:** Yuzhu Zhang, Yuchen Liu, Jing Liu, Tiande Liu, Hu Xiong, Wen Li, Xiaowei Fu, Fan Zhou, Shousheng Liao, Lu Fang, Bo Liang

**Affiliations:** ^1^ Department of General Surgery, The Second Affiliated Hospital of Nanchang University, Nanchang, China; ^2^ Department of Pathology, The Second Affiliated Hospital of Nanchang University, Nanchang, China

**Keywords:** immunotharapy, PD-L1, tislelizumab, S-1, GBC

## Abstract

Gallbladder cancer (GBC) and cholangiocarcinoma are common cancers of the biliary system and are associated with a poor prognosis. Surgery and chemotherapy provide limited benefit to patients with advanced biliary tract carcinoma. Novel immunotherapies and molecularly targeted therapies are more effective options; however, few patients benefit and drug resistance is a concern. Here, we report five cases of advanced GBC with either high programmed death-ligand 1 (PD-L1) expression or a high tumor mutation burden (TMB-H). The patients were treated with a combination therapy of tislelizumab and S-1. The tumors were effectively controlled in most patients. One patient developed immune-related pneumonia (irP) during treatment, which resolved after hormone therapy, and the patient underwent surgery. Tislelizumab and S-1 were administered again after surgery; however, recurrent irP required discontinuation, and the tumor progressed after drug withdrawal. These cases demonstrate that combined therapy of anti-programmed cell death protein-1 (PD-1) antibodies and S-1 is a safe and effective regimen with few side effects for GBC patients, especially for sensitive populations (patients with TMB-H, microsatellite instability, deficient mismatch repair, or high expression of PD-L1). To our knowledge, this is the first time that tislelizumab in combination with S-1 has been used to treat patients with advanced GBC.

## 1 Introduction

Gallbladder cancer (GBC) and cholangiocarcinoma (CHOL) are common cancers of the biliary system, with GBC accounting for 80–95% of all biliary tract carcinomas (BTCs) (1, 2). The 5-year overall survival (OS) of patients with BTC is below 5% (3). Surgical resection is the only curative treatment for BTC (4, 5), but only 10–15% of patients can undergo surgery because most patients are diagnosed with advanced disease or distant metastasis (6, 7). Chemotherapy is the most common adjuvant treatment for cancers of the biliary system. However, the objective response rate (ORR) of standard first-line chemotherapy (cisplatin plus gemcitabine [GC]) is only approximately 20% (8).

Immunotherapy and targeted therapy have shown good therapeutic promise for cancer in recent years (9, 10). The FDA (Food and Drug Administration) has approved targeted inhibitors of mutated isocitrate dehydrogenase 1 (IDH1), fibroblast growth factor receptor 2 (FGFR2), and casein kinase (CK2) for advanced or metastatic CHOL and durvalumab for locally advanced or metastatic BTC. However, few patients benefit from targeted therapy, and drug resistance is a concern (11–14). Therefore, the treatment of BTC remains challenging.

Here, we report our experience treating five GBC patients with high PD-L1 expression or high tumor mutation burden (TMB-H) who received a combination of tislelizumab and S-1. We present the comprehensive clinical evaluations and relevant histories of the patients and highlight the possible association between immunotherapy markers and efficacy. All patients achieved good therapeutic results and showed good treatment tolerance.

## 2 Case descriptions

### 2.1 Case 1

**Figure 1:**
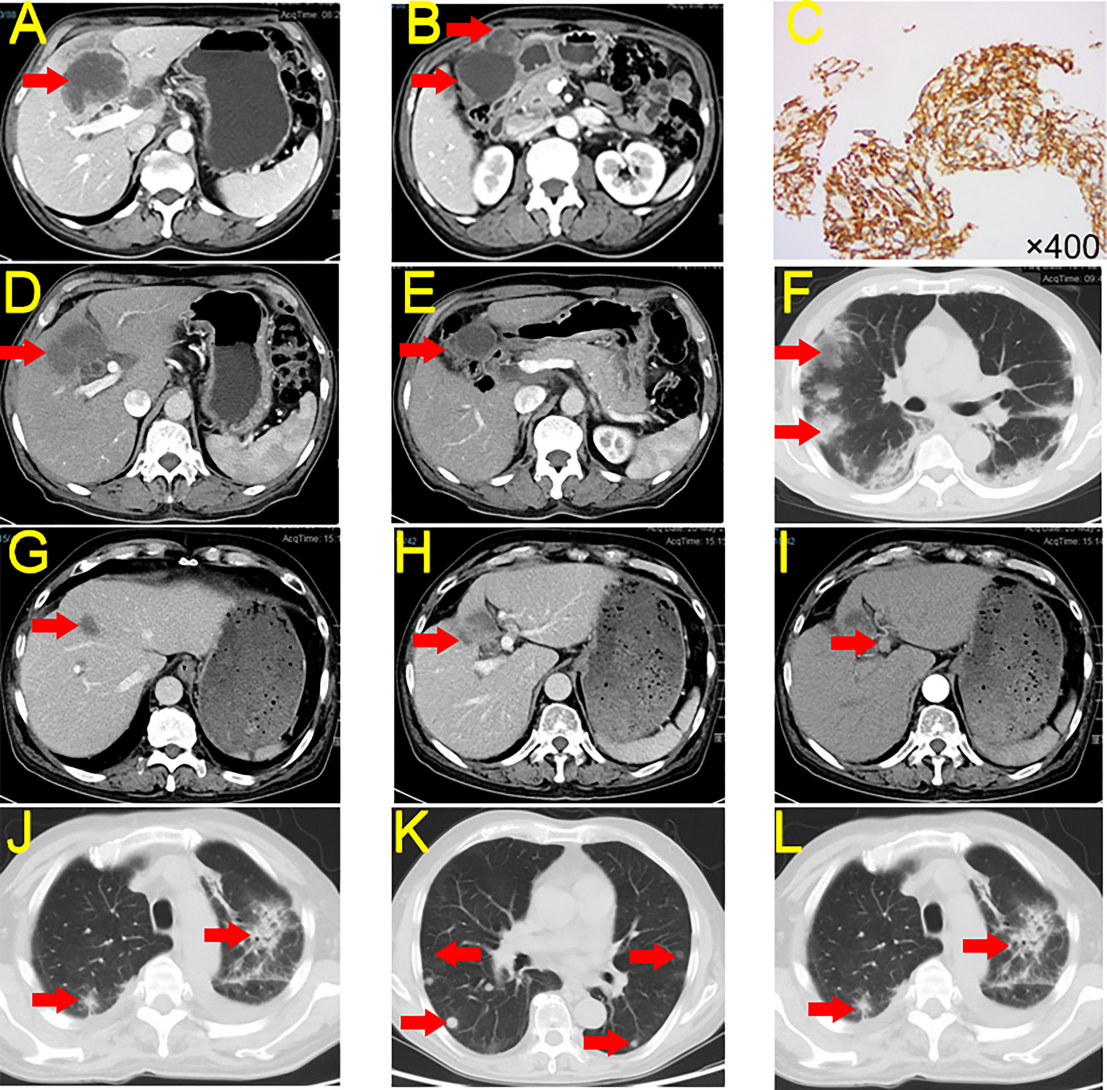
CECT and Immunohistochemistry for Case 1. **(A, B)** Tumor condition at first CECT scan. **(C)** Immunohistochemistry of PD-L1 expression. **(D, E)** Tumor conditions after seven cycles of treatment of tislelizumab + S-1. **(F)** irP after treatment. **(G, H)** The tumor shrank after continued immunotherapy, but the patient developed irP. **(I)** New lymph node metastasis. **(J)** irP developed during postoperative tislelizumab administration. **(K)** Lung metastasis. **(L)** irP after administration of nivolumab. CECT, contrast-enhanced computed tomography; PD-L1, Programmed Cell Death-Ligand 1; irP, immune-related pneumonia.

A 64-year-old man sought medical attention in September 2020 for marasmus. The physical examination showed no yellow pigmentation of the patient’s skin or sclera and no abdominal tenderness. Contrast-enhanced computed tomography (CECT) and positron emission tomography-computed tomography (PET-CT) of the abdomen showed a significant mass in the gallbladder and left hepatic region. Further, there were multiple metastatic nodules in the omentum and multiple metastases in the hilar and retroperitoneal lymph nodes (**Figures 1A, B**). CT-guided biopsy of the liver mass demonstrated poorly differentiated adenocarcinoma, and GBC with multiple intrahepatic and abdominal metastases were considered. The patient’s carbohydrate antigen 19-9 (CA19-9) was 188.49 U/mL (normal range, 0–37 U/mL), and his cancer antigen 125 (CA125) was 86 U/mL (normal range, 0–35 U/mL). The rest of the preoperative liver functional parameters were normal. We also performed next-generation sequencing (NGS) and the test report indicated a low tumor mutation burden (TMB-L) (1.76 Muts/Mb) and microsatellite stability (MSS). Immunohistochemistry demonstrated high expression of PD-L1 (Dako 22-C3, tumor proportion score (TPS) 90%, combined positive score (CPS) 90) in the tumor tissues (**Figure 1C**).

This patient had a complex disease course. He received seven cycles of tislelizumab + S-1 between October 2020 and February 2021. CECT indicated that the patient’s tumor shrank 89.37% (186.25 cm^3^ to 19.80 cm^3^, partial remission [PR]) and the abdominal metastases disappeared (**Figures 1D, E**). However, the patient developed immune-related pneumonia (irP) (**Figure 1F**) and had to discontinue immunotherapy but he continued S-1 treatment. After more than two months of meprednisone therapy, the patient’s pneumonia was in remission. Therefore, he restarted tislelizumab and S-1 (**Figures 1G, H**).

However, his CA19-9 levels continued to rise. After two cycles of treatment, CECT indicated that the primary tumor continued to shrink and the abdominal metastases had disappeared, but there were newly enlarged lymph nodes in the hilar area (**Figure 1I**). We did not identify any distant metastases on PET-CT. On May 2021, the patient underwent a laparoscopic cholecystectomy plus partial resection of liver S4b + 5 and regional lymph node dissection. The postoperative histopathological examination (HPE) report showed massive necrosis of the intrahepatic tumor tissue, no active tumor cells, and acute and chronic inflammatory cell infiltration. Individual atypical cells were observed in the liver tissue near the gallbladder. There was one positive lymph node in group 12, and no cancer cells were observed in the lymph nodes in groups 8, 9, 13, and 16 (tumour-node-metastasis(T4N1M1) stage: IVB).

Because of the lymph node metastases, the patient received tislelizumab + S-1 for two months after surgery. However, he again developed irP, so immunotherapy was discontinued, and he received S-1 monotherapy (**Figure 1J**). Six months after monotherapy, the patient began to develop lung metastases (**Figure 1K**). To avoid the development of irP, he switched to gemcitabine plus S-1 for treatment, but the tumor in the lung region continued to progress. Therefore, we administered three cycles of nivolumab. However, the tumor continued to enlarge and irP recurred (**Figure 1L**). As of February 2023, the patient was alive but more than two years after diagnosis, the tumor had not been controlled.

### 2.2 Case 2

**Figure 2:**
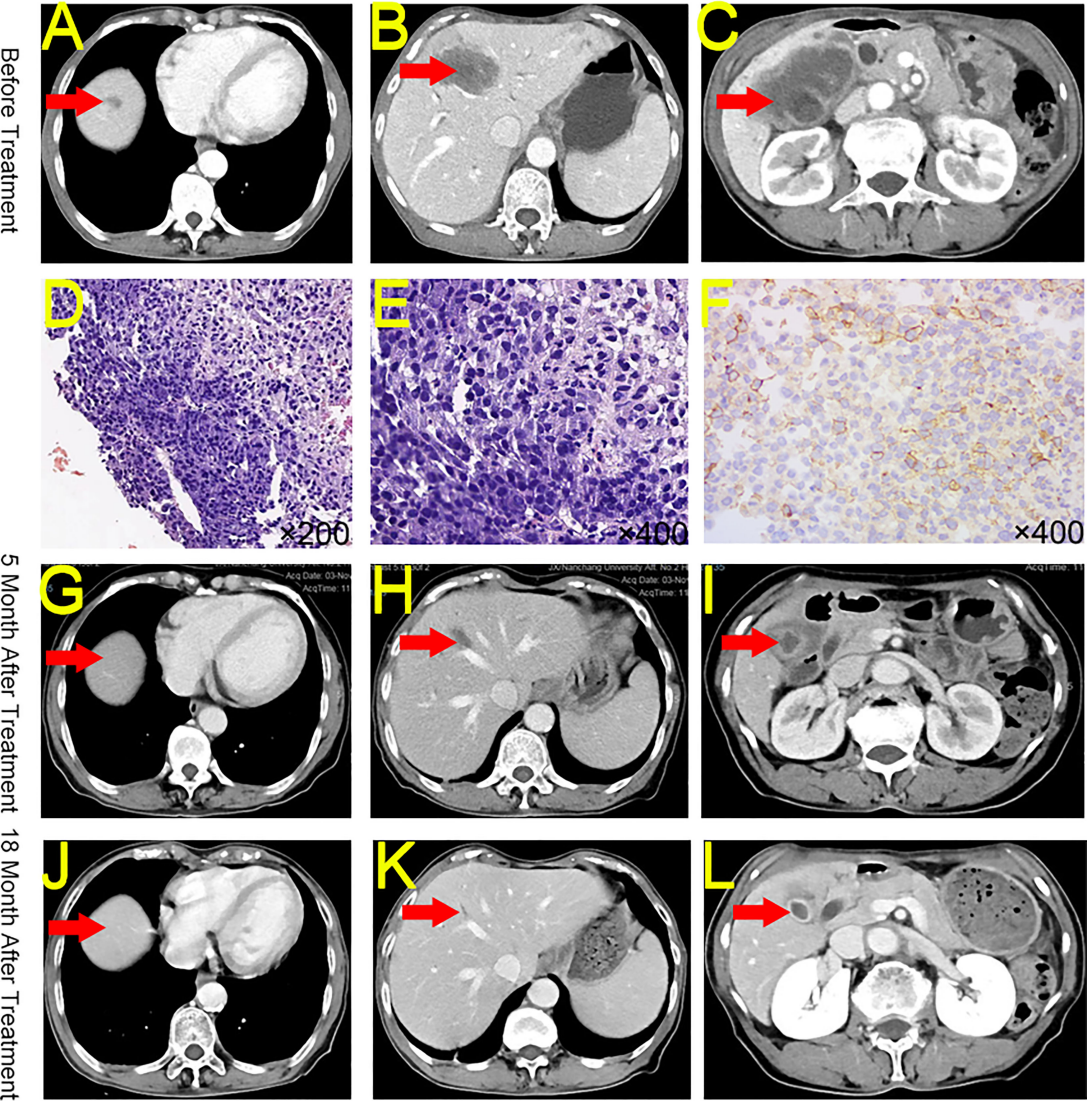
CECT, HE, and Immunohistochemistry for Case 2. **(A)** Intrahepatic mass at diagnosis. **(B)** A second intrahepatic mass at diagnosis. **(C)** Peri-gallbladder condition at diagnosis. **(D)** Hematoxylin and eosin staining of biopsy tissue (original magnification ×200). **(E)** Hematoxylin and eosin staining of biopsy tissue (original magnification ×400). **(F)** PD-L1 staining of biopsy tissue (original magnification ×400). **(G)** The reduction of the intrahepatic mass after 5 months of treatment. **(H)** Significant reduction in a second intrahepatic mass after 5 months of treatment. **(I)** Significant reduction in the peri-gallbladder mass after 5 months of treatment. **(J)** Persistent reduction of the intrahepatic mass after 18 months of treatment. **(K)** Persistent reduction in a second intrahepatic mass after 18 months of treatment. **(L)** Persistent reduction in the peri-gallbladder mass after 18 months of treatment. CECT, contrast-enhanced computed tomography; HE, hematoxylin and eosin; PD-L1, Programmed Cell Death-Ligand 1.

A 69-year-old woman sought medical attention in June 2021 for abdominal pain. The physical examination showed no yellow pigmentation of the patient’s skin or sclera and no abdominal tenderness. CECT showed gallbladder and intrahepatic region lesions, suggesting GBC with intrahepatic metastasis. In addition, the tumor had invaded the hilar vessels, and the patient was unable to undergo surgery (T4NxM1, IVB) (**Figures 2A-C**). Ultrasonography-guided liver biopsy confirmed liver invasive or metastatic poorly differentiated carcinoma (**Figures 2D, E**). The patient’s CA19-9 was 29.2 U/mL and her CA125 was 55 U/mL. The rest of the liver functional parameters were normal. NGS testing indicated TMB-H (16.48 Muts/Mb) and MSS. Immunohistochemistry indicated low expression of PD-L1 (Dako 22-C3, TPS 10%) in the tumor tissues (**Figure 2F**).

The patient received 26 cycles of tislelizumab + S-1 regularly between July 2021 and February 2023. The patient was offered curative surgery but she refused and chose to continue medication. As of November 2022, CECT indicated that the patient’s tumors shrank 98.28% (from 66.33 cm^3^ to 1.14 cm^3^, PR) (**Figures 2G-L**). Moreover, the patient’s CA19-9 and CA125 levels returned to normal.

### 2.3 Case 3

**Figure 3:**
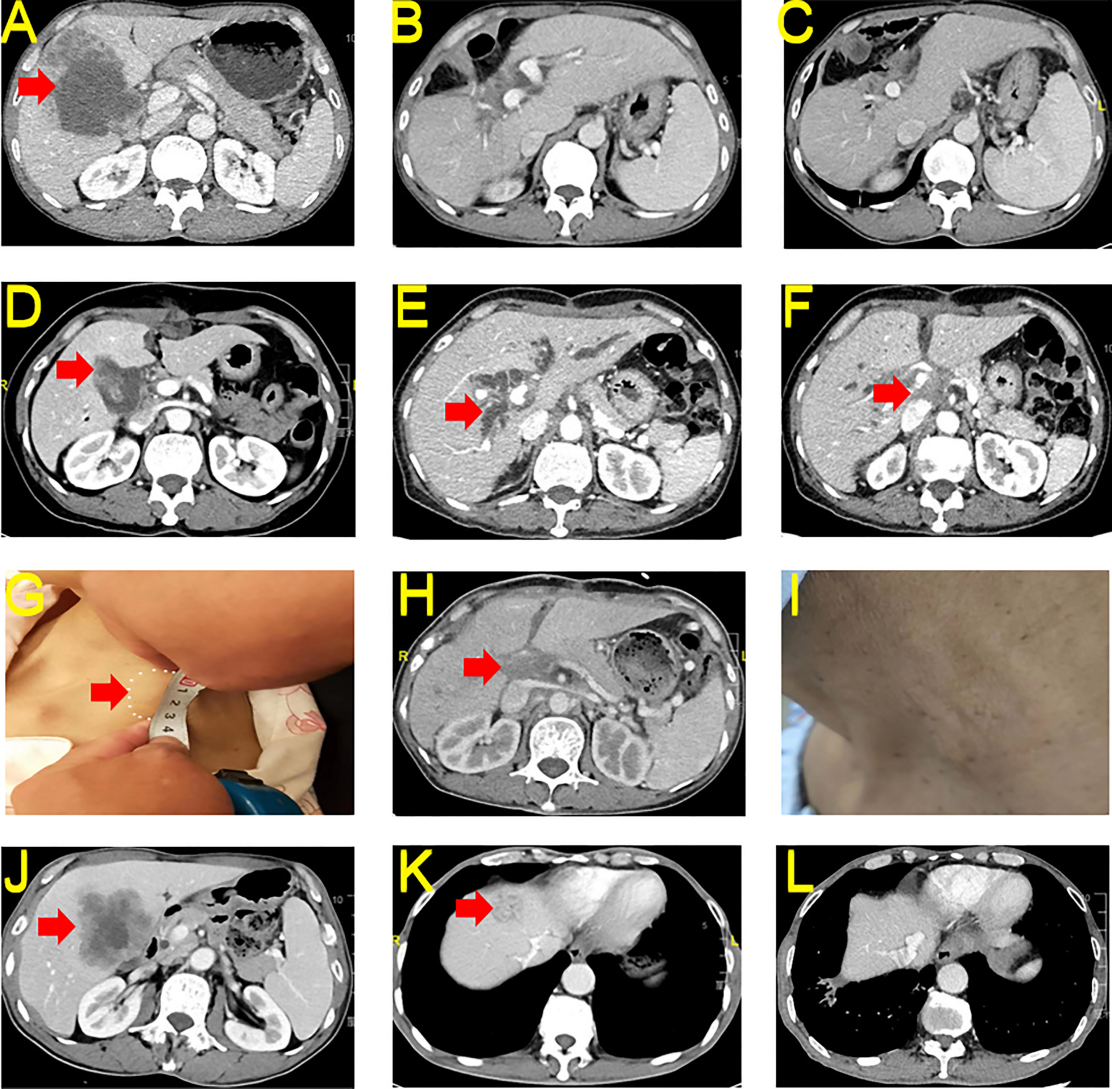
CECT and Macroscopy for Case 3-5. **(A)** Preoperative tumor conditions of case 3. **(B)** Postoperative 6 months reexamination of case 3. **(C)** Postoperative 18 months reexamination of case 3. **(D)** Preoperative tumor conditions of case 4. **(E)** Postoperative progression and bile duct dilatation of case 4. **(F)** Postoperative progression and hilar soft tissue effects of case 4. **(G)** Postoperative neck metastasis of case 4. **(H)** Hilar soft tissue shadow slightly reduced after immunotherapy in case 4. **(I)** Neck tumor disappeared after immunotherapy in case 4. **(J)** Preoperative tumor conditions of case 5. **(K)** Postoperative intrahepatic metastasis of case 5. **(L)** Intrahepatic metastases reduced after immunotherapy in case 5. CECT, contrast-enhanced computed tomography; HE, hematoxylin and eosin; PD-L1, Programmed Cell Death-Ligand 1.

A 55-year-old man sought medical attention in June 2021 for upper abdominal pain. The physical examination showed no yellow pigmentation of the patient’s skin or sclera and no abdominal tenderness. CECT showed space-occupying lesions in the gallbladder region, invasion of the right liver and right colon suggesting GBC with intrahepatic invasion, and invasion of the hepatic region of the colon (**Figure 3A**). The patient’s CA 19-9 was 274.81 U/mL, and CA 12-5 was 108.6 U/mL. The rest of the preoperative liver functional parameters were normal. The patient decided to undergo a cholecystectomy plus partial resection of liver S4b + 5 and regional lymph node dissection, a right hemicolectomy, a bile duct-jejunal ROUX-Y anastomosis, a right hemicolectomy and an ileum-colon anastomosis on June 9, 2021. The postoperative HPE report showed medium-low differentiated adenocarcinoma of the gallbladder and suggested that tumor-free margins (R0) was achieved (T4N1M0, IVA). NGS testing indicated TMB-H (17.93 Muts/Mb) and MSS. Immunohistochemistry indicated low expression of PD-L1 (Dako 28-8, TPS <1%) in the tumor tissues.

Due to the presence of liver and colon invasion and lymph node metastases in this GBC patient, the risk of postoperative recurrence was very high. The patient received 23 cycles of tislelizumab + S-1 regularly between June 2021 and October 2022. As of December 31, 2022, CECT showed no significant tumor recurrence (complete response (CR)) (**Figures 3B, C**), and the patient’s CA 19-9 and CA 12-5 levels returned to normal.

### 2.4 Case 4

A 53-year-old woman sought medical attention in August 2021 for a diagnosis of pathology suggestive of GBC after laparoscopic cholecystectomy one week earlier. The physical examination showed no yellow pigmentation of the patient’s skin or sclera and no abdominal tenderness. Healing surgical scars were visible on the patient’s abdomen. Pathology revealed a gallbladder tumor invading the serosa. CECT showed postoperative changes in the gallbladder region, and no metastases were observed in the liver. (**Figure 3D**) The patient’s CA 19-9 was 21.37 U/mL and CA 12-5 was 29.80 U/mL. The rest of the preoperative liver functional parameters were normal. The patient received a partial resection of liver S4b + 5 and regional lymph node dissection on August 9, 2021, after confirmation that there were no distant metastases. The HPE report showed medium-low differentiated adenocarcinoma of the liver tissue adjacent to the gallbladder. Cancer tissue metastasis was observed in 2 of 2 lymph nodes in group 8, 1 of 3 lymph nodes in group 12, and 1 of 6 lymph nodes in groups 13 and 16. We also observed a poking hole 2.0 cm below the xiphoid (T3N2M1, IVB).

Under the doctor’s care, the patient began receiving GS regimen chemotherapy after surgery. On January 14, 2022, the patient developed a hilar soft tissue shadow with intrahepatic bile duct dilatation (**Figure 3E**), and percutaneous transhepatic cholangial drainage was performed. On May 5, 2022, tumor progression developed, accompanied by an abdominal CECT revealing multiple enlarged lymph nodes in the hilar region and abdominal, and portal vein tumor thrombosis. (**Figures 3F, G**) An abnormal mass was palpable in the neck, and cervical metastasis of the tumor was considered. We performed NGS, and the report indicated TMB-H (7.1 Muts/Mb) and MSS. Immunohistochemistry indicated no expression of PD-L1 (Dako 28-8, TPS 0%) in the tumor tissues. The patient began receiving tislelizumab and S-1 treatment on June 3, 2022. As of December 31, 2022, CECT showed that the patient’s tumor shrank 76.45% (from 34.89 cm^3^ to 8.21 cm^3^, PR) and the patient’s neck mass disappeared. (**Figures 3H, I**).

### 2.5 Case 5

A 64-year-old male sought medical attention on May 12, 2022, for upper abdominal pain persisting for more than one month. The physical examination showed no yellow pigmentation of the patient’s skin or sclera and no abdominal tenderness. CECT revealed GBC with local intrahepatic invasion (**Figure 3J**). The patient’s CA 19-9 level was more than 700 U/mL, and CA 12-5 was 35.9 U/mL. The rest of the preoperative liver functional parameters were normal. The patient received a laparoscopy, a cholecystectomy plus partial resection of liver S4b + 5 and regional lymph node dissection, and a biliary-intestinal anastomosis after on May 19, 2022, after confirming there were no distant metastases. The postoperative HPE report suggested that R1 resection was achieved. The report also showed a gallbladder adenocarcinoma invading the gallbladder wall’s full thickness and surrounding liver tissue. In addition, nerve and hepatic arterial sheath invasions were found. No metastases were found in any of the 12 groups of lymph nodes (T4N0M0, IVA). The patient did not receive chemotherapy after surgery. However, the tumor recurred and was found on reexamination two months after surgery. CT showed intrahepatic metastasis (**Figure 3K**). Immunohistochemistry indicated a high expression of PD-L1 (Dako 28-8, TPS >60%) in the tumor tissues.

The patient began receiving tislelizumab and S-1 treatment on July 30, 2022. As of February 2023, he had received 10 cycles of treatment and CECT showed that the tumor shrank 97.55% (from 7.75 cm^3^ to 0.19 cm^3^, PR) (**Figure 3L**).

**Figure 4:**
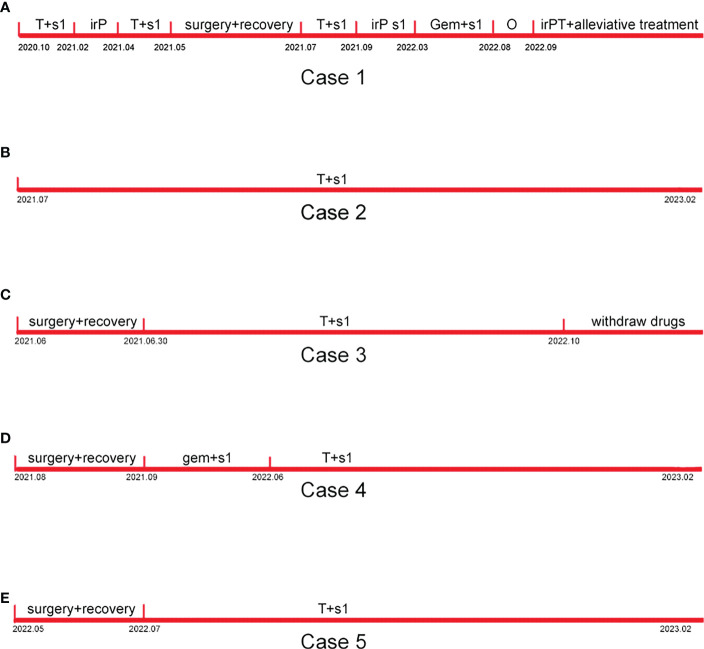
Timelines of Case 1-5. **(A)** The timeline of case 1. **(B)** The timeline of case 2. **(C)** The timeline of case 3. **(D)** The timeline of case 4. **(E)** The timeline of case 5.

The timelines of Cases 1–5 are shown in **Figure 4**.

## 3 Discussion

The FDA recently approved durvalumab combined with GC for adult patients with locally advanced or metastatic BTC based on the pivotal results of the TOPAZ-1 study, which showed that the combination of durvalumab plus GC prolonged OS (median OS 12.8 months vs. 11.5 months) and progression-free survival (PFS) (median PFS 7.2 months vs. 5.7 months) compared with chemotherapy alone (15). Unfortunately, few patients can benefit from this treatment (9, 16).

In the decade since immunotherapy became available for BTC, some case reports have shown good treatment outcomes. Lenvatinib plus anti-PD-1 treatment tends to improve survival in patients with advanced BTC after failure with GC (17); the median PFS and OS in patients receiving combination therapy were 4.0 months (95% confidence interval: 3.5–5.0) and 9.50 months (95% confidence interval: 9.0–11.0), respectively. However, 98.64% of patients reported adverse events (15). A phase I study based on 30 Japanese patients with unresected or recurrent biliary tract cancer showed anti-tumor activity for nivolumab as a monotherapy (median OS 5.2 months; median PFS 1.4 months) and in combination with GC (median OS 15.4 months; median PFS 4.2 months) (18). Another report showed that pembrolizumab monotherapy in BTC patients with PD-L1 expression in 1% or more of tumor cells or with tumor-infiltrating lymphocytes achieved responses in 4 out of 24 (17%) patients (19). Hepatic artery infusion chemotherapy (HAIC) in combination with immunotherapy also has high anti-tumor activity (20–22). One retrospective study pooled over 100 BTC patients treated *via* HAIC with the 3cir-OFF regimen, which is comprised of oxaliplatin, 5-fluorouracil, and folinic acid. The median PFS and OS of patients receiving this regimen were 9.8 months and 14.2 months, respectively (22). A study from China showed that tislelizumab was also observed to have anti-tumor activities in other solid tumors with an ORR ≥15% including nasopharyngeal carcinoma (43%), MSI-H/deficient mismatch repair (dMMR) solid tumors (19%), non-small cell lung cancer (18%), gastric cancer (17%), hepatocellular carcinoma (17%), and melanoma (15%) (23).

Some reports have shown that S-1 alone is effective and well-tolerated in patients with advanced GBC (24–26). Three patients achieved partial or complete remission after S-1 monotherapy, suggesting that S-1 could be used as an alternative therapy if standard first-line chemotherapy drugs were not tolerated. Results from a recent large trial of Japanese patients undergoing BTC resection showed a significant increase in both three-year OS (77.1% vs. 67.6%) and three-year relapse-free survival (62.4% vs. 50.9%) for patients treated with S-1 compared to those who did not receive it (27). Results from another trial investigating the efficacy of combined chemotherapy with S-1 and cisplatin in the treatment of metastatic or recurrent BTC showed an overall ORR of 30% and a median OS of 8.7 months (28). In addition, the synergistic effect of nab-paclitaxel and GC has shown good antitumor efficacy and controllable safety (29, 30). A phase II clinical trial (ClinicalTrials.gov identifier: NCT02392637) showed that nab-paclitaxel combined with GC prolonged OS (19.2 months vs 11.7 months) compared with GC alone (31).

Several studies suggest that high PD-L1 expression, TMB-H, MSI, and deficient mismatch repair (dMMR) are potential biomarkers for immunotherapy (32–35) and that tumors with these biomarkers may show higher ORR (36–38). However, stereotactic body radiotherapy combined with nivolumab or pembrolizumab showed good survival benefits and acceptable toxicity in patients with intrahepatic cholangiocarcinoma with TMB-L, MSS, mismatch repair-proficient (pMMR), and no PD-L1 expression (expression level < 1%) (39, 40). Further, some patients without high PD-L1 expression respond to immune and targeted combination therapy. Some BTC patients with mutated sites have low PD-L1 expression (TPS ≤10%) but still achieve significant clinical benefits from targeted combined anti-PD-L1 therapy (41–43). Of note, none of these previously published cases mention other biomarkers such as TMB, MMR, or MSS.

Given the superiority of immune and targeted combination therapy shown by published studies, several clinical trials on immunotherapy and targeted therapy in patients with GBC are ongoing (ClinicalTrials.gov identifiers: NCT04003636, NCT05239169, NCT04333927, NCT04308174, NCT04466891, NCT04183712, and NCT04211168).

The TOPAZ-1 study showed that combination therapy did not have significantly different survival benefits based on PD-L1 expression (15). However, other biomarkers were not considered when performing grouping. Another clinical trial administered nivolumab alone for patients with refractory BTC and found that patients with high PD-L1 expression had a significantly longer PFS than those with low PD-L1 expression, and all patients who responded to treatment had dMMR (44). Therefore, we believe that although the overall benefit is limited, immunotherapy combined with chemotherapy will achieve better clinical outcomes in sensitive populations (patients with TMB-H, MSI, dMMR, or high expression of PD-L1). However, it is still unclear whether combination therapy benefits these sensitive patients more than immunotherapy alone.

Because the patient in Case 1 developed irP, we changed the treatment from tislelizumab to nivolumab. However, his irP recurred, and his tumor continued to progress. Therefore, it appears that switching medications after the patient develops immune-related adverse events does not decrease the severity of the side effects. In addition, while waiting to start nivolumab, the patient developed drug resistance. In Case 2, after the medication was effective and the surgical criteria were met, the patient chose to continue medication instead of surgery. Although the patient showed good clinical results, we still cannot determine whether it is feasible to forgo surgery after response to combination therapy. Further, if the patient opts to continue treatment, determining how to judge whether there is tumor activity by CECT and how long to discontinue the drug after the development of adverse events remains a problem. In Case 3, considering the patient’s high risk of postoperative recurrence, we administered the combination of tislelizumab and S-1 to the patient postoperatively. Currently, the patient has not experienced a relapse for 19 months. The patients in cases 4 and 5 developed tumor progression after surgery. After which they received tislelizumab + S-1 and the tumor shrank. Combining the results from the three patients (Cases 3, 4, and 5), we suggest that timely postoperative drug administration for patients with medication indication may reduce tumor progression and the recurrence rate.

Only the patient in case 1 developed irP; the rest of the patients had no immune-related adverse events. Four of the five patients profiled in this study underwent surgery, and all of them recovered smoothly with no significant postoperative complications. In our study of immunotherapy for GBC, we found only one case of irP, but our team also found immune-related liver damage and immune-related myocarditis in immunotherapy for liver cancer. Deaths occurred in these two patients. This study has several limitations. First, the samples size of our study is too small to draw general conclusions. Thus, further studies are required to establish the effectiveness of the combined treatment with tislelizumab plus S-1. Second, we did not recheck the PD-L1 and TMB status of patients which go on to form metastases in continued immunotherapy. We think it may be beneficial to test the PD-L1 and TMB status of patients’ specimens if patients show resistance to anti-PD-1 immunotherapy.

## 4 Conclusions

The five cases we report show that the combination of anti-PD-1 and S-1 therapy is a safe and effective treatment option for patients with advanced GBC and may be more useful for those with a high expression of immunotherapeutic markers (PD-L1, TMB, etc.).

## Data availability statement

The original contributions presented in the study are included in the article/supplementary material. Further inquiries can be directed to the corresponding authors.

## Ethics statement

Written informed consent was obtained from the individual(s) for the publication of any potentially identifiable images or data included in this article.

## Author contributions

SL, LF, and BL contributed to the conception and design of the study. SL, LF, and BL contributed to the administrative support of the study. FZ, JL, SL, LF, and BL contributed to the provision of study materials for patients. YL contributed to the collection and assembly of data for the study. YZ and YL contributed to the data analysis and interpretation of the study. All authors contributed to the article and approved the submitted version.
